# Factors influencing implementation of a survivorship care plan—a quantitative process evaluation of the ROGY Care trial

**DOI:** 10.1007/s11764-016-0562-3

**Published:** 2016-08-01

**Authors:** Belle H. de Rooij, Nicole P. M. Ezendam, Kim A. H. Nicolaije, M. Caroline Vos, Johanna M. A. Pijnenborg, Dorry Boll, Roy F. P. M. Kruitwagen, Lonneke V. van de Poll-Franse

**Affiliations:** 1 CoRPS—Center of Research on Psychology in Somatic diseases, Department of Medical and Clinical Psychology, Tilburg University, Tilburg, The Netherlands; 2 The Netherlands Comprehensive Cancer Organisation, Utrecht, The Netherlands; 3 Department of Obstetrics and Gynecology, Gynecologic Cancer Center South, Elisabeth-TweeSteden Hospital, Tilburg and Waalwijk, The Netherlands; 4 Department of Obstetrics and Gynecology, Catharina Hospital, Eindhoven, The Netherlands; 5 Department of Gynecology and GROW - School for Oncology and Developmental Biology, Maastricht University Medical Center, Maastricht, The Netherlands; 6 Division of Psychosocial Research and Epidemiology, The Netherlands Cancer Institute, Amsterdam, The Netherlands

**Keywords:** Survivorship care plan, Implementation, Information provision, Gynecologic cancer

## Abstract

**Purpose:**

The aim of this study is to investigate the factors that influence implementation of Survivorship Care Plans (SCPs) in the intervention arm of the ROGY Care trial by (1) assessing the level of SCP receipt in the ROGY Care trial and (2) identifying patient- and provider-level factors that influence SCP receipt.

**Methods:**

Between 2011 and 2015, a pragmatic cluster randomized-controlled-trial was conducted on the effects of automatically generated SCPs. Endometrial (*N* = 117) and ovarian (*N* = 61) cancer patients were allocated to ‘SCP care’, as provided by their SCP care providers (*N* = 10). Associations between SCP receipt (self-reported SCP receipt and actually generated SCPs), patient-factors (socio-demographic-, clinical-, and personality factors), and care provider factors (profession and a-priori motivation regarding SCP provision) were tested in univariate analysis. The odds ratios of factors influencing self-reported SCP receipt were estimated with a multivariate regression model.

**Results:**

Of all patients in the SCP care arm (*N* = 178), SCPs were generated by the care provider for 90 % of the patients and 70 % of the patients reported that they had received an SCP. Patients with older age, ovarian cancer, type D (distressed) personality, and patients that completed the questionnaire a longer period of time after the SCP consult were more likely to report no SCP receipt.

**Conclusions:**

SCP receipt was influenced by patient- but not care-provider factors.

**Implications for cancer survivors:**

Certain patient groups were less likely to report SCP receipt. Whether all patients are in need of an SCP, requires further investigation. If they do, more efforts need to be made towards the implementation of SCPs.

## Introduction

In 2006, the American Institute of Medicine (IOM) and the Dutch Health Council advocated Survivorship care plans (SCPs) as a standard of care for all cancer patients [1, 2]. An SCP is a formal document that is handed to the patient and includes a record of all care received, important disease characteristics of the patient, short- and long-term effects of the treatments received and information for supportive care services [1]. SCPs aim to promote cancer survivors’ follow-up care and outcomes [1]. However, since the IOM’s recommendations, implementation and dissemination of SCPs in clinical practice have been low and inconsistent [3–6].

Our recently published study of the pragmatic cluster-randomized ROGY Care trial [7] was accompanied by an editorial declaring the need for more attention to implementation of SCPs [8]. The ROGY Care study contributes to the small number of clinical trials that have evaluated the effects of SCPs on patient reported outcomes [9–11]. Published SCP trials could not draw definite conclusions on SCP effectiveness and highlighted the complexity of SCP implementation [7, 9–11]. It is therefore recognized that, alongside investigations of the effectiveness of SCPs, we need to understand how SCPs were implemented and compare implementation strategies between SCP trials [8, 12]. The pragmatic nature of the ROGY Care trial provides the unique opportunity to evaluate implementation of SCPs in clinical practice.

Evaluations of intervention implementation often include a measure of fidelity—that is, the degree to which an intervention was delivered as intended [13]. The implementation fidelity of SCPs (i.e., the content of the SCP, the coverage of patients that receive SCPs and the frequency of SCP receipt) is expected to have an impact on survivors’ outcomes [8]. Subsequently, poor implementation fidelity of SCP care in clinical trials would diminish the observed effects of SCPs, leading to an underestimation of true SCP effectiveness [13].

Little is known about the factors that influence implementation of SCPs. Current evidence is predominantly based on qualitative studies that focus on system- and organizational level factors that influence SCP implementation, including organizational resources, adequate (electronic) systems, templates, and training for SCP use [3, 14–19]. A few observational studies have revealed that patients with lower age, non-white race, higher income, higher educational level, better than fair health status, and patients that participated in a trial, more often reported receipt of an SCP [4, 19–21]. However, generalizability of these observational studies is limited due to patient selection bias. Furthermore, in these studies, only self-reported receipt of SCPs by patients was available [4, 19–21]. To our knowledge, no SCP effectiveness randomized controlled trials have yet examined the factors that influence implementation of SCPs.

The ROGY Care trial provides longitudinal quantitative data on a wide range of patient-level and provider-level factors, along with both objective and self-reported implementation outcomes of SCPs in routine Dutch clinical practice. Patient-level factors include demographic, clinical, and personality characteristics, and provider-level factors include demographic characteristics, profession, and a-priori opinions regarding SCPs. Understanding factors that promote or inhibit successful implementation of SCPs in the ROGY Care trial can support future implementation of SCPs [8, 12].

The aim of the current study is to investigate factors that influence implementation of SCPs in the ROGY care trial by (1) assessing the level of SCP receipt and (2) identifying patient- and provider-level factors that influence SCP receipt.

## Methods

### Design

Between April 2011 and October 2015, the pragmatic cluster-randomized ROGY Care trial was conducted to evaluate the impact of an automatically generated SCP on gynecological cancer patient and health care provider reported outcomes. In the South of the Netherlands, 12 hospitals were randomized to either ‘usual care’ or ‘SCP care’. After initial diagnosis, all endometrial and ovarian cancer patients were invited to participate in the study. Patients were invited with a letter, informed consent form, and questionnaire, sent to the patient by their own gynecologist [22, 23]. Follow-up questionnaires were sent directly to the patient at 6, 12, 18, and 24 months after diagnosis*.* Patients, but not care providers, were blinded to trial assignment. The ROGY Care trial was centrally approved by a Medical Research Ethics Committee, as well as by each participating center [22]. The trial design has been described in detail elsewhere [22]. The present study describes the results of implementation fidelity in the intervention arm.

### Patients and care providers

Participants include 117 newly diagnosed endometrial and 61 ovarian cancer patients that were in the intervention arm of the ROGY Care trial and completed the first questionnaire, and their ten SCP care providers (i.e., gynecologists, gynecologic oncologists, and oncology nurses) in the six hospitals of the intervention arm. A follow-up questionnaire was sent to the patients 12 months after diagnosis. Follow-up questionnaires were returned by 68 % (*N* = 79) of the endometrial and 57 % (*N* = 35) of the ovarian cancer patients. Patient exclusion criteria (i.e., undergoing palliative care or unable to complete a Dutch questionnaire) [22] were minimal to maximize generalizability [24]. All care providers of the intervention arm (*N* = 10) completed a questionnaire before the start of the trial [25].

### Implementation of SCP care

In the hospitals that were allocated to ‘SCP care’, all care providers attended an instruction evening. The care providers were instructed to provide an SCP to patients after diagnosis and to provide an updated SCP during follow-up visits if applicable (i.e., when there were changes in the cancer, treatment, or oncology provider). In addition, care providers were instructed to send a copy of the SCP to the patient’s primary care physician [26]. Practical guidelines were given on the components of the SCP that should minimally be discussed with each patient during the SCP consult (i.e., diagnosis, prognosis, treatment(s), and most important, side-effects) and how often the SCP should be discussed (shortly after diagnosis and during follow-up visits after 6, 12, 18, and 24 months). Care providers in the SCP care arm were instructed to provide the first SCP at the consultation where the results of histopathology and (adjuvant) treatment plan were discussed, mostly 7–14 days after the operation or biopsy. Because of the pragmatic approach, care providers in the SCP care hospitals were free to choose whether the gynecologist/gynecologic oncologist, and/or oncology nurse provided the SCP, fitting their clinical practice [22]*.* No other care providers (i.e., medical oncologists or radiotherapists) were involved in the trial because they do not use the registration system through which SCPs were generated.

SCPs could be automatically generated through the web-based ‘Registrationsystem Oncological GYnecology’ (ROGY), which is used by all participating oncology providers in both arms since 2006. For each patient, a detailed registration is made in a uniform way, including tumor stage and grade, treatment, comorbidity, complications, follow-up, and information about the involved specialists (e.g., gynecologist/gynecologic oncologist, medical oncologist, and radiotherapist). For the ROGY Care trial, an application was built in ROGY that enables automatic generation of an SCP combining patient and disease data from ROGY. Care providers could generate an SCP by pressing a button in ROGY. This button was only visible for the care providers in the intervention arm.

### Survivorship care plan

The SCP was based on the Dutch translation of IOM’s SCP template [27], adjusted to the local situation [28] by a group of gynecologists/gynecologic oncologists, oncology nurses, a radiotherapist, medical oncologist, primary care physician, and patients [22]. Texts of the SCP were based on pilot-tested patient education material from the Dutch Cancer Society. In addition, the SCP was pilot-tested on patients with a low/intermediate educational level to ensure that the SCP was understandable.

The SCP consisted of a tailored treatment summary including information on diagnostic tests, type of cancer, stage, grade, treatment(s) (type, date, and specialist), and contact details of the hospital and specialists. The treatment summary contained explanatory notes of the clinical information provided and visual representations of affected organs and cancer stage. In addition, the SCP contained a tailored follow-up care plan, including detailed information on the most common short- and long-term effects of the treatments received, effects on social and sexual life, possible signs of recurrence and secondary tumors, and information on rehabilitation, psychosocial support, and supportive care services [22].

### Measures

#### SCP receipt

Receipt of SCPs was assessed by the number of patients for whom SCPs were generated, the number of patients that reported having received an SCP, the number of patients for whom follow-up SCP(s) were generated, and the number of patients that reported having received a follow-up SCP. The number of patients of whom (first and follow-up) SCPs were generated was obtained from ROGY. ROGY recorded whether the SCP was generated for the patient by a care provider. Whether the patients actually received the SCP was based on self-report, by asking ‘Did you receive a survivorship care plan?’. No further explanation about the SCP was given in the questionnaire to avoid feelings of disadvantage in the control arm. Follow-up SCP receipt was assessed by follow-up questionnaires (‘How often did you receive a survivorship care plan?’), on 6 and 12 months after diagnosis. Patients that reported (first or follow-up) SCP receipt while no SCP was generated in ROGY were allocated to ‘reported no (first or follow-up) SCP receipt’, because it was not possible to receive an SCP when not generated.

#### Patient factors

Age, socioeconomic status (SES) and clinical data, such as cancer type, cancer stage, and date of diagnosis, were obtained from the Netherlands Cancer Registry (NCR). The NCR routinely collects data on newly diagnosed cancer patients in all hospitals in the Netherlands [29]. SES was based on postal code of the residence area of the patient, combining aggregated individual fiscal data on the economic value of the home and household incomes [30]. SES was categorized into low, medium, or high.

Shortly after diagnosis, a first questionnaire was sent to the patient to assess partner status, the number of comorbidities and Type D personality. Partner status was dichotomized (having a partner vs. not having a partner). The number of comorbidities was assessed by the adapted self-administered comorbidity questionnaire (SCQ) [31]. Type D (distressed) personality is defined as the joint tendency towards negative affectivity (e.g., worry, irritability, and gloom) and social inhibition, and has previously been associated with lower perceived receipt of information in cancer patients [32]. Type D personality was assessed by the Type D scale (DS14) [33].

In a follow-up questionnaire 12 months after diagnosis, health literacy was measured by one item of the 5-point Chew’s scoring scale (‘How confident are you by filling out medical forms?’) [34]. Low health literacy was defined as being somewhat, a little or not at all confident filling out medical forms, medium health literacy was defined as being quite confident filling out medical forms, and high health literacy was defined as being very confident filling out medical forms [34]. Furthermore, the time between SCP consult and completion of questionnaire was calculated by the difference in weeks between first treatment received (obtained from the NCR) and the date of filling out the first questionnaire.

#### Care provider factors

The primary care provider (i.e., gynecologist or gynecologic oncologist) that was in charge of the SCP care of the patient, was registered in ROGY. In three out of six hospitals in the intervention arm, the provision and discussion of the SCP was delegated to an oncology nurse. Age, sex, and motivation of the care providers regarding SCP provision and opinion about SCP benefit were measured by a questionnaire among all care providers before the start of the trial [25]. Motivation regarding SCP provision (‘How motivated are you to start using the SCP?‘) and opinion about SCP benefit (‘To what extent do you expect the SCP to affect the patient positively?’) were measured on a 10-point scale (strongly disagree—strongly agree).

### Statistical analysis

Statistical analyses were conducted using Statistical Analysis System (SAS) version 9.4. (SAS Institute, Cary, NC, 1999). Means with standard deviations (SD) were used to describe normally distributed continuous variables, medians, and interquartile ranges (25th–75th) to describe not normally distributed variables and frequencies (N) with percentages (%) to describe categorical variables. All patient- and care provider-level factors influencing SCP receipt were assessed in univariate analysis, using independent samples *t* tests for normally distributed continuous variables, Mann—Whitney U Test for not normally distributed continuous variables and Chi^2^-tests for categorical variables. For categorical variables with an expected count less than five, Fisher’s exact tests were used. In the main analysis, the dependent variable was SCP receipt as reported by the patient. In additional analyses, dependent variables were generated SCPs, reported receipt of follow-up SCPs and generated follow-up SCPs. Independent variables were patient- or provider-level factors. Independent variables with a significance level greater than 0.05 were entered into a multivariable logistic regression model using a forward selection method. For each selected independent variable, the odds of SCP receipt as reported by the patient was estimated (SCP received vs. no SCP received). A significance level of 0.05 was used.

## Results

### SCP receipt

**Fig. 1:**
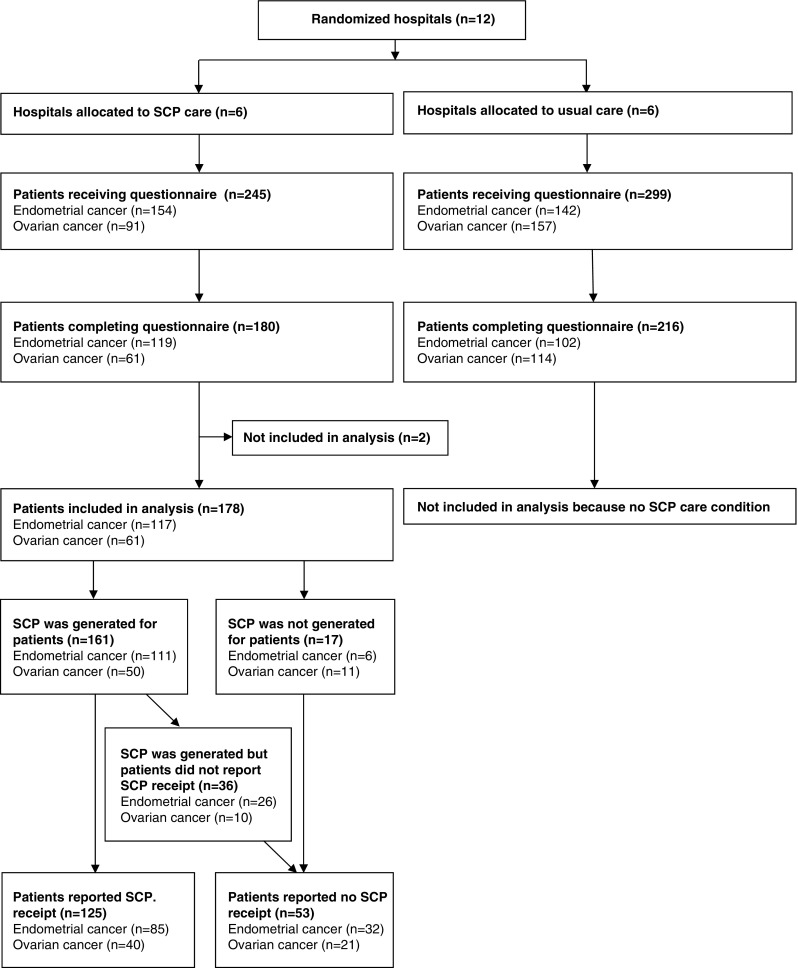
Flow diagram of patients included in analysis and (first) SCP receipt in the ROGY care trial

Of all 178 patients in the intervention arm of the trial, an SCP was *generated* for 90 % of the patients (*N* = 161). From the patients for whom an SCP was *generated*, 78 % (*N* = 125) *reported* receipt of an SCP (Fig. 1).

### Patient and SCP care provider factors related to SCP receipt

In univariate analysis, patients who *reported* first SCP receipt were significantly younger (65 years versus 70 years, *p* < 0.01) and less often had Type D personality (15 % vs. 31 %, *p* = 0.02) compared to patients that *reported* no first SCP receipt (Table 1). In endometrial cancer patients, first SCPs were more often received by patients with an advanced FIGO stage (Table 1). No SCP care provider factors were associated with first SCP receipt (Table 2). Multivariate analysis showed that older age, having ovarian cancer vs endometrial cancer, having Type D personality versus no type D personality and completion of the questionnaire a shorter period of time after the SCP consult were all independently associated with a lower chance of *report* of first SCP receipt (Table 3).

**Table 1:** Patient factors of reporting first SCP receipt

	SCP Received (*N* = 125)	SCP Not received (*N* = 53)	*P* value
Age, mean (SD)	64.7 (10.2)	70.4 (8.6)	<0.01
Cancer type, *N* (%)			
Endometrial	85 (68)	32 (60)	0.33
Ovarian	40 (32)	21 (40)	
Endometrial cancer			
FIGO stage, *N* (%)			
I	68 (80)	32 (100)	0.051
II	6 (7)	0 (0)	
III	9 (11)	0 (0)	
IV	2 (2)	0 (0)	
Treatment type			
Surgery only	53 (64)	16 (50)	0.07
Radiotherapy	25 (30)	16 (50)	
Chemotherapy	5 (6)	0 (0)	
Ovarian cancer			
FIGO stage, *N* (%)			
I	15 (38)	6 (28)	0.43
II	6 (15)	1 (5)	
III	14 (35)	9 (43)	
IV	5 (12)	5 (24)	
Treatment type			
Surgery only	10 (26)	5 (25)	0.96
Chemotherapy	29 (74)	15 (75)	
SES, *N* (%)			
Low	20 (18)	12 (25)	0.14
Medium	41 (36)	21 (45)	
High	53 (47)	14 (30)	
Partner			
Yes	93 (76)	38 (73)	0.66
No	29 (24)	14 (27)	
Health literacy^a^, *N*(%)			
Low	38 (45)	7 (28)	0.15
Medium	40 (47)	13 (52)	
High	7 (8)	5 (20)	
Type D personality, *N* (%)			
Yes	18 (15)	15 (31)	0.02
No	105 (85)	34 (69)	
Comorbidities, *N* (%)			
0	17 (14)	4 (8)	0.09
1	32 (26)	21 (43)	
>1	75 (60)	24 (49)	
Weeks between SCP consult and questionnaire, median (25th–75th)	10.7 (7.0–14.6)	11.2 (7.0–15.9)	0.43

^a^ *Low* being somewhat, a little or not at all confident filling out medical forms; *medium* being quite confident filling out medical forms; *high* being very confident filling out medical forms. Health literacy was unknown for *n* = 68

*P* values are based on independent samples *t* tests for continuous variables and Chi^2^-tests/ Fisher’s exact tests for categorical variables

All percentages stated are column percentages

**Table 2:** SCP provider factors of reporting first SCP receipt

	SCP received (*N* = 125)	SCP not received (*N* = 53)	*P* value
Hospital, *N* (%)			
1	27 (22)	10 (19)	0.98
2	14 (11)	6 (11)	
3	23 (18)	10 (19)	
4	36 (29)	17 (32)	
5	10 (8)	4 (8)	
6	15 (12)	6 (11)	
SCP Care provider, *N* (%)			
Gynecologist/ OG	63 (50)	27 (51)	1.00
Oncology nurse	62 (50)	26 (49)	
Age SCP provider, mean(SD)			
	43.5 (5.0)	43.2 (5.2)	0.75
Gender SCP provider, N (%)			
Male	9 (7)	4 (8)	1.00
Female	116 (93)	48 (92)	
Motivation regarding SCP provision, mean (SD)			
Range 0–10	8.1 (0.7)	8.0 (0.7)	0.94
Opinion about SCP benefit, mean (SD)			
Range 0–10	7.4 (1.1)	7.4 (1.1)	0.90

*P* values are based on independent samples *t* tests for continuous variables and Chi^2^-tests/ Fisher’s exact tests for categorical variables

**Table 3:** Odds ratio’s (OR) of first SCP receipt versus no first SCP receipt

	SCP received versus not received (*N* = 146)		
	OR	95 % CI	*P* value
Age, per 10 years	0.35	0.20–0.57	**<0.01**
Cancer type,			
Endometrial	1.00 (ref)		
Ovarian	0.31	0.12–0.83	**0.02**
Type D personality,			
Yes	0.28	0.11–0.73	**<0.01**
No	1.00 (ref)		
Comorbidities			
0	1.00 (ref)	0.07–1.65	
1	0.37	0.21–4.82	0.22
>1	1.12		0.89
Time between SCP consult and questionnaire, per week	0.95	0.90–1.00	**0.04**

Candidate variables for multivariate regression were all patient factors (age, cancer type, FIGO stage, treatment type, socio-economic status, health literacy, Type D personality, number of comorbidities and number of weeks between SCP consult and questionnaire) and care provider factors (hospital, SCP Care provider, age, gender, motivation regarding SCP provision and opinion about SCP benefit). Candidate variables with a significance level higher than 0.05 were entered into a multivariate regression model using a forward selection method. Selected variables were entered into a separate multivariate regression model in order to include all patients in the model for whom data was available on selected variables.

Follow-up SCPs were *reported* as received by 21 % (*N* = 27) of the patients (data not shown). *Reported* receipt of follow-up SCPs was associated with a hospital (*p* < 0.01) and having an oncology nurse as SCP provider compared to a gynecologist/oncologic gynecologists (31 vs. 10 %, *p* < 0.01) (not tabulated). No patient factors were associated with receipt of follow-up SCPs.

Additional analyses showed that first SCPs were more often *generated* for endometrial compared to ovarian cancer patients (95 vs. 82 %, *p* < 0.01). Follow-up SCPs were also more often *generated* for endometrial compared to ovarian cancer patients (24 vs. 11 %, *p* = 0.04) and more often for ovarian cancer patients who had surgery only compared to ovarian cancer patients who also had chemotherapy (40 vs. 9 %, *p* = 0.04) (not tabulated).

In addition, 36 patients (20 %) for whom a first SCP was generated (N_total_ = 161) did not report receiving an SCP. These patients were significantly older compared to patients who *reported* first SCP receipt (71 [SD 8.0] vs. 65 [SD 10], *p* < 0.01) (not tabulated).

## Discussion

In this study of endometrial and ovarian cancer survivors in the intervention arm of the ROGY Care trial, first SCPs were generated for 90 % of the patients and reported as received by 70 % of the patients. Follow-up SCPs were reported as received by 21 % of the patients. Patient factors, including being older, having ovarian cancer and having a Type D personality were independently associated with a lower chance of perceived receipt of first SCPs, while having an oncology nurse as care provider was associated with higher perceived receipt of follow-up SCPs.

To our knowledge, this is the first study that examines patient and care provider factors associated with SCP receipt in a trial. The self-reported SCP receipt in our trial is substantially higher compared to the coverage ranging between 24 and 58 % in observational studies [35]. This is probably related to the ease with which the SCP could be automatically generated through ROGY by clicking a button. However, due to the pragmatic nature of the trial, 100 % dissemination of SCP receipt was not attained.

Interestingly, we found that a considerable group of patients reported no SCP receipt while an SCP was generated. These patients were older on average compared to patients who did report receiving an SCP. There are two possible explanations for this finding: SCP receipt may have been underreported by older patients due to a recall bias, or during consultation care providers decided more often not to hand over the SCP to older patients. In line with our findings, younger age has previously been associated with higher self-reported SCP receipt in observational studies [20, 21]. This has formerly been explained by a higher need for instructions for follow-up care in younger cancer patients [20]. In addition, recall bias may explain lower self-reported SCP receipt in older patients.

Cancer type has also previously been associated with differences in SCP receipt. In two observational studies, patients with more common types of cancer, including breast-, prostate-, lung-, and colorectal- cancer reported SCP receipt more often compared to patients with less common types of cancer such as melanoma and gynecological cancers [20, 21]. It is possible that this is related to the fact that SCP templates are less available for less common types of cancer [3, 36]. SCP receipt in endometrial and ovarian cancer patients specifically has not previously been investigated. Brothers and coworkers’ SCP trial in gynecological cancer patients did not examine how many SCPs were actually received in the intervention arm [9]. Our study showed that SCPs were more often generated for, and more often reported as received by, endometrial cancer patients compared to ovarian cancer patients. Maybe, SCP care providers perceive more barriers to providing information to cancer patients with worse prognosis. This is in accordance with literature showing that health care providers are often reluctant to provide information on late effects in order to prevent disproportionate fear in the patient [37]. Further, ovarian cancer patients less often received follow-up SCPs when they had chemotherapy compared to surgery only. This could be explained by the fact that during chemotherapy, treatment of the patient is scheduled for follow-up visits at the medical oncologist instead of the gynecologist/oncologic gynecologist [25]. Medical oncologists were not involved in our trial and therefore did not provide SCPs.

Besides younger age and cancer type, other studies found that higher SES is associated with higher perceived SCP receipt [20, 21]. In addition, higher health literacy has been associated with higher perceived information provision [38]. Although we did not find statistically significant differences, our data suggest positive trends between SES and perceived SCP receipt, and health literacy and perceived SCP receipt.

To date, no patient personality factors have been studied in relation to SCP receipt. Our study shows that patients with a Type D personality (a combination of negative affect and social inhibition), were more likely to report no SCP receipt. Patients with this personality type have the tendency to experience increased negative emotions and tend not to share these emotions because of fear of rejection or disapproval [33]. In this study, 19 % of the patients had a Type D personality, which is comparable to 21 % in the general population [33]. Other studies have shown that cancer patients with a Type D personality are less likely to report receipt of both oral and written information [32]. SCP receipt in patients with a Type D personality may have been underreported. This may be due to negative emotions they experience towards medical information [32], or because SCP care providers may be more reluctant to provide SCPs for patients that are more inhibited and less likely to ask for information. Future research should explore whether information needs are lower among patients with a Type D personality and consequently whether lower provision of SCPs for patients with a Type D personality is desired.

A minority of the patients in our study received a follow-up SCP. We found that follow-up SCP receipt, but not first SCP receipt, was higher in hospitals where SCP care was delegated to an oncology nurse. This was mainly due to the presence of one oncology nurse that provided SCP care for a large number of the patients in our analysis. Therefore, the generalizability of this finding is questionable. Moreover, we could not adjust for the patients’ need of an updated SCP (i.e., when there were changes in the cancer, treatment, or care provider), which could have biased our results. However, previous studies also suggest that oncology nurses promote successful implementation of SCPs [18, 39]. Consistently, prior results from the ROGY Care trial showed that oncology providers in our study (i.e., gynecologists, gynecologic oncologists and oncology nurses) prefer oncology nurses to provide SCPs in their practice [25]. Therefore, for improved implementation of follow-up SCPs, delegation of SCP care to an oncology nurse is recommended. However, oncology nurses did not provide first SCPs more often compared to gynecologists/oncologic gynecologists in our study.

Another suggestion to improve implementation of follow-up SCPs may be that the follow-up SCPs’ content is tailored to the information needs of the patient during follow-up. In our trial, follow-up SCPs only differed from first SCPs when there were substantial differences in the treatment or care provider. If other information is provided in a follow-up SCP than the first SCP, care providers would probably be more prone to provide follow-up SCPs. Further, it would probably be helpful for the care provider if a reminder is sent when a follow-up SCP needs to be delivered.

A strength of the current study is the trial design, in which large numbers of patient- and SCP care provider factors were measured along with longitudinal objective and subjective measures of SCP receipt. In addition to self-reported receipt of SCPs that has been examined in previous literature, we were able to examine whether an SCP was generated or not. This revealed new insights into, for instance, a possible recall bias of reported SCP receipt related to older age, and more certainty about factors influencing actual SCP receipt including cancer type and Type D personality.

In order to maximize the generalizability of our trial results, the ROGY Care trial is characterized by a pragmatic approach; exclusion criteria for patient inclusion were limited and oncology providers were free to choose how the SCP provision was integrated in clinical practice. Despite the pragmatic nature of the trial, however, adherence to SCP provision by the care providers was probably higher than we would expect in clinical practice outside a trial setting [3, 36]. For instance, SCP care providers in our trial frequently received reminders for patient inclusion and providing an SCP if not done so yet. This is reflected by a relatively long period between SCP provision and completion of the questionnaire by the patient. Our findings may therefore not be fully generalizable to everyday routine clinical practice.

Limitations of our study include the uncertainty of our measure of SCP receipt; although we were able to objectively examine whether an SCP was generated through ROGY, we are not sure whether the SCP was handed over to the patient. Therefore, we have to rely on self-report of the patient. However, our results suggest that the self-reported assessment of SCP receipt may have been affected by recall bias in older patients. Besides that, independent from age, patients who completed the questionnaire a longer period of time after the SCP consult were more likely to report no SCP receipt. A delay in completion of the questionnaire was either caused by a longer time needed for the gynecologist to include a patient in the study and sending the questionnaire, or by the patient taking a longer time before filling out the questionnaire after receiving the questionnaire. Either way, this may indicate a recall bias of self-reported SCP receipt. Future studies should therefore aim to include a more reliable measure of SCP receipt in the study, for instance by sending a questionnaire shortly after SCP receipt in order to prevent recall bias. Alternatively, SCP receipt could be recorded by the care provider, but this may result in over-report of SCP receipt due to a social desirability bias.

The level of implementation fidelity of SCPs in the ROGY Care trial is expected to have an influence on the observed effectiveness of SCPs. When no 100 % coverage of SCP receipt in the intervention arm is attained, a comparison between the intervention and control arm (intention to treat analysis) may result in an underestimation of SCP effectiveness on patient reported outcomes. Therefore, a per protocol analysis could provide a more accurate estimation, by only comparing patients who reported SCP receipt to all patients in the usual care arm [7]. The current study shows, however, that SCP receipt may have been underreported due to recall bias. Subsequently, only patients who accurately remembered SCP receipt (i.e., because of younger age or more extensive discussion of SCP by the care provider) were included, which may result in an overestimation of SCP effectiveness. Therefore, both types of analysis require careful interpretation. It remains debatable whether a per protocol analysis based on actually generated SCPs instead of self-reported SCP receipt would better reflect SCPs effectiveness in the ROGY Care trial.

Our findings can support future implementation of SCPs in clinical practice if widespread implementation is decided upon, or future clinical trial research. Disparities in SCP care could be reduced by paying particular attention to older patients and patients with ovarian cancer, who appear to less often receive SCPs. In addition, care providers providing SCPs should pay particular attention to patients with a Type D personality, as they experience more negative emotions towards medical information and are not likely to ask for information themselves. However, the question arises whether all patients are in need of information as provided in an SCP. For instance, whether patients with a distressed personality benefit from SCP receipt instead of unnecessarily accumulating psychosocial distress requires further investigation [7]. Possibly, more personalized SCPs (i.e., modules fitting individual patients’ information needs) could promote information provision for cancer survivors in clinical practice.
